# A Medical Causerie

**Published:** 1916-04-08

**Authors:** 


### A MEDICAL CAUSERIE.

It will be interesting to observe how the Royal Colleges will work with the Central War Committee in the medical recruiting scheme. The suggestion came from the War Committee, and has been under discussion for some weeks. The primary motive was provided by the position of the staffs of the London hospitals. The Committee naturally desired to know to what extent these could be further reduced without definite prejudice to the work of the hospitals and to the interests of the civil population. Such information necessarily involved some rather delicate questions, and it was felt that these could be more safely explored by representatives of the Royal Colleges than by a Committee containing many members whose activities are concerned with general practice rather than with hospital work. The hospital staffs, of course, consist of men who are associated with one or other of the Colleges, and thus might be expected to defer to the Colleges when perhaps they might resent inquiries from a body having a less definite professional status. The new arrangement has not been easily accomplished, but now it is in existence there is every hope that it will work smoothly. There are memories of former antagonisms between the Royal Colleges and the British Medical Association. Especially is this true in reference to the College of Physicians, but it would, indeed, be a mean and petty thing that such memories should hinder hearty co-operation in the presence of a great national emergency. At the same time, one cannot deny that there are opportunities for friction unless the sphere of the Colleges in this matter is plainly defined. It must be clearly understood that their function is to be purely an advisory one, and that the position of the War Committee in relation to the Government is not in any degree invaded. The notion that the Colleges are now to supersede the War Committee or to review its decisions is quite unfounded. They may make representations to the Committee and to the Government through the Committee, but here their function ends. To suppose that the work which the Committee has carried on can be transferred to an entirely new authority now it is half done is little short of absurd, as is also the idea that the profession would extend to the Colleges a confidence it has denied to the Committee. Whatever the virtues of the Colleges, it is notorious that they represent only a limited field of the general interests of the profession, and their governing councils, more especially that of the College of Physicians, possess a very restricted base of sympathy and support.

The medical wants of the Army present opportunities which seem to have attracted attention outside, as well as inside, the four corners of the Empire. This would seem at least to be indicated by an announcement which recently appeared in one of the American medical journals to the effect that the Allies are likely shortly to need 2,500 more English-speaking doctors. It is added that several well-paid vacancies exist in South Africa, and that probably some of these positions will be open to Americans. No one can foretell what will be the necessities of the future, but if all goes well with the enrolment scheme the Director-General's needs are likely to be met by British practitioners. The opposition to the scheme has certainly diminished now that a definite statement of numbers has been made and it is realised that unless the proposal is carried through the doctors will have to deal with the War Office without the protective medium of a professional organisation. The success of the scheme will mean that the profession has of itself, and apart from War Office co-operation or pressure, met all the medical wants of the fighting forces, and the prestige of such an accomplishment is not lightly to be despised.

It is one of the functions of members of Parliament to find opportunities for indignation, but one may doubt whether much sympathy will be felt for the gentleman who recently denounced the authorities for declining to employ bone-setters in the treatment of injured and partially disabled soldiers. Considered purely from the administrative side the suggestion is an impossible one, for where is the line to be drawn if once the limit of the "registered medical practitioner" is passed? Admittedly the whole question of unqualified practice is a difficult one, and it has social and economic relations as well as professional aspects. But the idea that the War Office can safely add to its numerous responsibilities the choice of saying Aye or No to unqualified persons offering to undertake medical treatment is indeed an ingenuous one. Because a member of Parliament believes himself to have been cured by a bone-setter, therefore bone-setters should be set to work on British soldiers, is a form of argument which would lead to strange results. The badgered officials would have every variety of quack at their doors.

Medical columns in lay papers are not easy to conduct with success, but they have their function and value. They must, however, be kept on sure ground and are more suited for elementary truths than for speculative enterprises. Recently, in what used to be called the leading journal, a "medical correspondent" has been finding proof that civilisation is "going the pace" in the returns which show that in spite of the war the consumption of sugar has not diminished. Our legislators are urged to take note of this fact as a sign of the times and as an index of the whipped-up activities of the nation. Hence, we are warned, the days of reaction will be hard days, disease incidence may be expected to rise and the average expectation of life to fall. These are large conclusions, and the dietetic experts are likely to recognise that there is a new Richmond in the field.

